# Intact Rapid Facial Mimicry as well as Generally Reduced Mimic Responses in Stable Schizophrenia Patients

**DOI:** 10.3389/fpsyg.2016.00773

**Published:** 2016-05-31

**Authors:** Natalya Chechko, Alena Pagel, Ellen Otte, Iring Koch, Ute Habel

**Affiliations:** ^1^Department of Psychiatry, Psychotherapy and Psychosomatics, Medical School, RWTH Aachen UniversityAachen, Germany; ^2^Jülich-Aachen Research Alliance—Translational Brain Medicine, JülichAachen, Germany; ^3^Institute of Psychology I, RWTH Aachen UniversityAachen, Germany

**Keywords:** EMG, rapid facial mimicry, Simon task, dual-task, corrugator supercilii and zygomaticus major muscle

## Abstract

Spontaneous emotional expressions (rapid facial mimicry) perform both emotional and social functions. In the current study, we sought to test whether there were deficits in automatic mimic responses to emotional facial expressions in patients (15 of them) with stable schizophrenia compared to 15 controls. In a perception-action interference paradigm (the Simon task; first experiment), and in the context of a dual-task paradigm (second experiment), the task-relevant stimulus feature was the gender of a face, which, however, displayed a smiling or frowning expression (task-irrelevant stimulus feature). We measured the electromyographical activity in the corrugator supercilii and zygomaticus major muscle regions in response to either compatible or incompatible stimuli (i.e., when the required response did or did not correspond to the depicted facial expression). The compatibility effect based on interactions between the implicit processing of a task-irrelevant emotional facial expression and the conscious production of an emotional facial expression did not differ between the groups. In stable patients (in spite of a reduced mimic reaction), we observed an intact capacity to respond spontaneously to facial emotional stimuli.

## Introduction

Emotional processing deficits, including a reduced ability to understand and express facial emotions, contribute to the pathophysiology of schizophrenia. It has been suggested that patients with schizophrenia are less accurate in facial expression of affective messages, showing reduced facial action responsivity across emotions and generalized performance deficits in emotion recognition (for review see e.g., Mandal et al., 1998; Trémeau, 2006). Some studies have also indicated a trait-like deficit in spontaneous facial activity of schizophrenia patients (Gaebel and Wolwer, 1992; Walker et al., 1993; Varcin et al., 2010).

Exposed to particular facial expressions, people spontaneously mirror them with similar facial expressions (Dimberg et al., 2000; Wild et al., 2001). This phenomenon is known as rapid facial mimicry, which facilitates empathic feeling and social functioning, including the establishment of interpersonal relationship and understanding of other minds (Baron-Cohen, 2005). Schizophrenia, on the other hand, is associated with deficits in socio-emotional and communicative abilities. Investigations of spontaneous facial expression in schizophrenia, therefore, are of great theoretical and clinical relevance.

Previous studies have evaluated spontaneous facial expression deficits from emotion-eliciting interviews recorded on videotape (Gaebel and Wolwer, 1992), or home movies featuring children who later developed schizophrenia (Walker et al., 1993), or by means of electromyography (EMG) during passive viewing of emotional faces (Varcin et al., 2010). The number of such studies, however, is not only very small, but their results are also limited owing to the lack of standardized approaches. For instance, none of the mentioned studies controlled for effects of generally reduced emotional expression (i.e., flat affect) on rapid facial mimicry in schizophrenia. To eliminate this shortcoming from our work, we sought to investigate stimulated spontaneous facial reactions by means of standardized and previously evaluated stimuli in healthy controls in EMG tasks as described by Otte et al. (2011a,b). In the Simon paradigm (Otte et al., 2011a) and in the context of a dual task (Otte et al., 2011b), conscious mimic responses to compatible and incompatible stimuli were measured by EMG onset latency in the corrugator supercilii and zygomaticus major muscle regions. In both tasks, the interaction effect of simultaneous implicit perception of emotional faces on conscious production of facial expressions was used to quantify the level of rapid facial mimicry.

Applying these two tasks to both healthy controls and patients with schizophrenia, we hypothesized a reduced compatibility effect in schizophrenics due to deficits in automatic response to implicitly processed facial expressions. We also expected to see a generally reduced mimic response in patients as measured by the overall reaction time of the zygomaticus and corrugator muscles.

## Materials and methods

### Participants

Twenty patients with paranoid schizophrenia and fifteen healthy controls were recruited at the Department of Psychiatry, Psychotherapy, and Psychosomatics, RWTH Aachen University. All patients were outpatients, and stable at the time of the study. Five patients had to be excluded from data analysis because of technical errors and artifacts. The final sample for the Simon task comprised 15 patients (9 men) and the final sample in the dual task had 15 patients (10 men). For both tasks, there were 15 healthy controls, of whom nine were male.

Further demographic and clinical characteristics of the participants are outlined in Tables 1, 2.

**Table 1:** **Sociodemographic characteristics of the groups and illness-related data for patients who participated in the Simon task**.

	**Patients**	**Controls**	**Group comparisons**
Age (years)	38.04 ± 10.11	39.81 ± 10.9	n.s.
Mean education (years)	11.82 ± 1.47	11.85 ± 1.68	n.s.
Mean parental education (years)	9.54 ± 1.83	10.27 ± 1.94	n.s.
Processing speed (TMT-A, s)	25 ± 6.25	24.61 ± 10.99	n.s.
Cognitive flexibility (TMT-B, s)	46.73 ± 24.22	44.92 ± 16.68	n.s.
Verbal intelligence (MWT_B, hits)	24.82 ± 7.25	30.69 ± 3.47	n.s.
Crystallized general intelligence (GCR, hits)	105.07 ± 12.71	112.69 ± 12.7	n.s.
Regensburger *Word Fluency* Test (RWT, hits)	57.27 ± 9.45	65.00 ± 12.96	n.s.

*MWT-B, Multiple-Choice Vocabulary Intelligence Test, version B (Lehrl, 1989); TMT-A/B, Trail Making Test, version A and B (Reitan, 1958); WMS-R Wechsler memory test (Härtling et al., 2000); Regensburger Word Fluency Test (RWT; Aschenbrenner et al., 2001)*.

**Table 2:** **Sociodemographic characteristics of the groups and illness-related data for patients who participated in the dual task**.

	**Patients**	**Controls**	**Group comparisons**
Age (years)	40.24 ± 11	39.81 ± 10.9	n.s.
Mean education (years)	12.0 ± 1.41	11.85 ± 1.68	n.s.
Mean parental education (years)	9.23 ± 2.15	10.27 ± 1.94	n.s.
Processing speed (TMT-A, s)	24.77 ± 5.02	24.61 ± 10.99	n.s
Cognitive flexibility (TMT-B, s)	46 ± 22.59	44.92 ± 16.68	n.s.
Verbal intelligence (MWT-B, hits)	25.92 ± 7.42	30.69 ± 3.47	n.s.
Crystallized general intelligence (GCR, hits)	106.85 ± 16.71	112.69 ± 12.7	n.s.
Regensburger *Word Fluency* Test (RWT, hits)	58.38 ± 10.08	65.00 ± 12.96	n.s.

*MWT-B, Multiple-Choice Vocabulary Intelligence Test, version B (Lehrl, 1989); TMT-A/B, Trail Making Test, version A and B (Reitan, 1958); WMS-R Wechsler memory test (Härtling et al., 2000); Regensburger Word Fluency Test (RWT; Aschenbrenner et al., 2001)*.

The diagnosis of schizophrenia was made by the treating physicians according to the ICD-10. Most patients were treated with second generation antipsychotics. Four patients received olanzapine, three clozapine, three quetiapine, and one aripiprazol as a monotherapy. Two patients received combination therapy with clozapine and aripiprazole or quetiapine and aripiprazole, while two others were treated with a first generation antipsychotic (flupentixol depot). One patient was unmedicated for 3 weeks.

The mean time since first clinical admission was 10 (±6.7) years. A clinical interview and medical history review helped determine the stability of the patients' current clinical condition. The control group comprised 15 healthy subjects matched for age, gender, and parental education. Participants with current co-morbid Axis I and Axis II disorders [according to SCID, German version (Wittchen et al., 1996)], with past or current neurological disorder or other medical illnesses with impact on brain functioning, were not included. Disorders of the central nervous system, consumption of illegal drugs or alcohol within the last 2 years were additional exclusion criteria for both groups. We did not include any participants in the control group with manifested psychiatric disorder in close relatives. All participants were right-handed (Oldfield, 1971). The healthy participants were recruited by means of advertisements.

All participants signed the informed consent form prior to the study, which was conducted in concordance with the Declaration of Helsinki and approved by the Institutional Review Board of the Medical Faculty, RWTH Aachen University.

### Stimuli and apparatus

The applied stimuli were collected prior to the study and tested experimentally for both tasks (Otte et al., 2011a,b). Twenty-four professional actors from local theater schools had been asked to help create an emotional expression database. The actors were instructed and trained on the six basic emotions and subsequently videotaped. The videos were analyzed using the FACS coding system to ensure that the appropriate muscles had been used. In addition, the videos were rated by 69 students on the basis of affect, clarity, and realism of the emotions and emotional expressions, with only those with high ratings (90% or higher) in all categories having been used. Static images of emotional expressions at their peak were extracted to use in the current experiment, which included eight stimuli, showing two male and two female actors, each with either a happy or angry facial expression.

### Paradigm

All subjects participated in two separate experimental tasks, a stimulus-response compatibility paradigm (Simon task) and a dual-task paradigm, with the order of tasks being counterbalanced across participants. All experiments were carried out in the building of the University Hospital Aachen in rooms that were largely shielded from electrical noise sources. Between the tasks, there was a break to allow facial musculature to relax. Each task with EMG took 20 min, with the stimuli being presented full screen on a 19-inch monitor (35 × 25 cm) at a distance of ~70 cm from the participants. Both experiments were executed using MATLAB 2010, including the psychtoolbox 3.0.8 (Brainard, 1997; Pelli, 1997).

#### Stimulus-response compatibility paradigm (Simon task)

Participants were instructed to produce a happy or angry facial expression as promptly as possible in response to either a male or a female face, independent of the emotional expression of the face. In 50% of the trials, the presented and the to-be-produced facial expressions were compatible, and they were incompatible in the rest of the trials. The stimulus person's gender (female vs. male) and the required facial response (smile vs. frown) were counterbalanced across participants. To counterbalance the gender effects, half of the female and male participants were instructed to smile at female images, whereas the other half were instructed to smile at male images.

**Figure 1:**
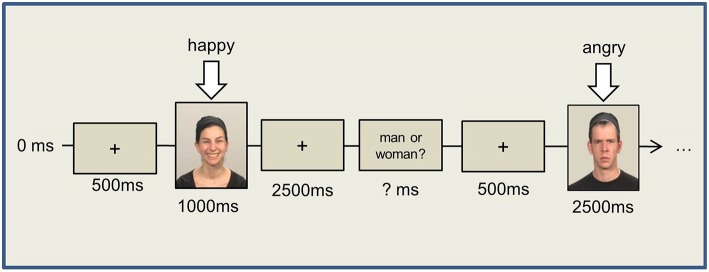
**Simon Task**.

The experiment itself began with a white fixation cross in the center of the screen. After another 500 ms, an image appeared on the screen depicting either a female or a male face with either a “happy” or an “angry” expression (see Figure 1). The image was visible for 1000 ms, after which the fixation cross reappeared. The participants had another 2500 ms following stimulus offset to respond and to relax their facial muscles again. To ensure the participants were indeed paying attention to the task, a question appeared following the fixation cross asking whether the stimulus just displayed was a woman or a man. Participants were instructed to answer pressing the answer button. The response time, however, was not restricted.

We presented four experimental blocks, each containing 40 randomized trials with an equal number of happy/angry expressions and male/female images, with intermittent breaks. Prior to the experimental blocks, 10 training trials were performed to check the correctness of the EMG signal and the participants' response patterns.

After the button press, another fixation cross was shown for 500 ms, followed by the next stimulus.

#### Dual task

For Task 1, pictures showing two male and two female faces were used as stimuli (S1), with the faces differing also in emotional expression (i.e., smiling or frowning, resulting in eight different pictures). Participants responded to these stimulus pictures (i.e., S1) by button press (left and right “ctrl” keys, R1) at the end of the dual-task trial. In Task 2, high- and low-pitch tones (500 and 1200 Hz) of 100 ms duration were presented via headphones. Task 2 was to produce a facial expression in response to the tone (i.e., R2, either smile or frown) as promptly as possible. The mapping of tone (high vs. low, S2) and the required facial response (smile vs. frown, R2) was counterbalanced across participants.

**Figure 2:**
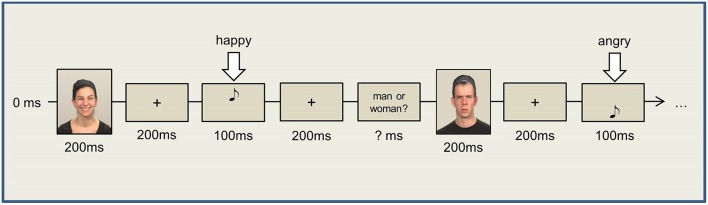
**Dual Task**.

The trial started with a white fixation cross (1000 ms) in the center of the screen, following which a picture from Task 1 (male or female face) appeared on the screen for only 200 ms to ensure immediate encoding. The picture was followed (SOA 200 ms) by a tone from Task 2 (i.e., the facial expression production task), with the participants being instructed to promptly produce either a smile or a frown in response to the tone. After a fixed interval of 2500 ms (starting with S2 onset), a stimulus prompted the response to the first task i.e., a question appeared on the screen asking whether the shown picture was that of a man or a woman. The next trial started 500 ms after manual R1 (please refer to Figure 2). Two experimental blocks of 80 trials each were presented, with different picture and tone stimuli as well as their combinations (i.e., eight different face stimuli and two different tones) being presented with equal frequency in each block. Consequently, S1–R2 compatible and incompatible trials occurred equally often. The trial sequence, however, was random and varied across participants.

Given the demanding nature of the tasks, we allowed participants to relax their facial muscles after 40 trials of each block. Prior to the experimental blocks, each participant performed 10 training trials for each single task separately and 20 training trials for the dual-task procedure.

### EMG recording and signal extraction

Facial EMG activity was recorded using a portable biosignal recorder (Varioport-B) by Becker Meditec. Miniature facial electrodes (Ag/AgCl electrodes with a diameter of 13 mm, and a contact site of 5 mm) were placed above respective muscle regions on the left side of the face, using synapse conductive electrode cream. Fridlund and Cacioppo (1986) defined the zygomaticus major as the main muscle used for smiling and the corrugator supercilii as the main muscle used for frowning. The EMG signal was passed through an analog digital transformer and was visualized in Variograf 4.68. The data were sampled at a rate of 256 Hz, and passed through a low pass filter at 100 Hz. Before extracting reaction times from the EMG signal, the signal was passed through a 10th order Butterworth low pass filter with a filter cut-off frequency of 70 Hz.

The extraction of reaction time occurred offline after the data were filtered, rectified, and organized into separate trials of 4.5 s (beginning to end, for each trial).

A marker was set before stimulus onset, with the first 1000 ms before this marker (i.e., stimulus onset) being used as a baseline. From this baseline, we computed a signal threshold, which was the mean of this baseline +2 SD + 10 μV. The 10 μV was added to remove artifacts such as muscle tension or muscle twitches. From stimulus onset, we determined the muscle onset latency by using a contour follower (as suggested by Fridlund and Cacioppo, 1986) with a 200 ms window. This mean signal had to exceed the set threshold for at least 50 ms to be recorded as an actual muscular reaction. The first time value of a muscle response (following stimulus onset) that exceeded this threshold was recorded as the muscle onset latency, hence reaction time. We determined baselines and reaction times for each muscle and trial separately to avoid differences in the EMG signal strength between the two muscles.

### Analysis of behavioral data

The program SPSS (version 18.0) was used for analyses. Reaction time was defined as the time between stimulus presentation and the crossing of the individually defined baseline in terms of a significant muscular tension compared to activity at rest.

We computed error percentages based on responses of the wrong muscle, double responses (response in both muscles), and missing responses. For RT measures, we removed all errors as described above as well as all trials after an error. We also excluded trials in which participants responded erroneously in terms of gender discrimination.

A Kolmogorov–Smirnov test was carried out to check normal distribution, and all data were normally distributed. We performed a multifactorial ANOVA, with the level of significance set at *p* < 0.05.

In the tasks, compatibility between presented and to-be-produced facial expressions (compatible vs. incompatible), *muscles* (zygomaticus major vs. corrugator supercilii), and *group* (controls vs. patients) were the independent variables. The dependent variables were RT and error rate. RT was measured as EMG onset latency, and errors included no reactions, reactions using the incorrect muscle, or reactions in which responses were measured in both muscles.

## Results

### Stimulus-response compatibility paradigm (Simon task)

We subjected the data to a three-factor analysis of variance (ANOVA) with *compatibility* and *muscle* as repeated measures variables and *group* as an independent factor. The ANOVA yielded a significant main effect of *compatibility* [*F*_(1, 14)_ = 28.448, *p* < 0.001, η^2^ = 0.70]. The descriptive statistics showed that responses in incompatible trials were slower (mean ± standard error: 460 ± 30 vs. 520 ± 33 ms for compatible and incompatible trials, respectively), revealing a compatibility effect of 60 ms.

The main factor *group* indicated a trend [*F*_(1, 14)_ = 3.818, *p* = 0.077, η^2^ = 0.26] toward slower overall muscle activity among patients, with the overall muscle activity being 534 ± 46 vs. 445 ± 30 ms (mean ± standard error) for patients and healthy controls, respectively. No further significant effects were found. Thus, the observed compatibility effect was similar for both muscles [*muscles* × *congruency* interaction: *F*_(1, 14)_ = 0.009, *p* = 0.927, η^2^ = 0.01, 62 vs. 59 ms for the zygomaticus major and corrugator supercilii, respectively] and both groups [*group* × *congruency* interaction: *F*_(1, 14)_ = 0.157, *p* = 0.700, η^2^ = 0.01, 66 vs. 54 ms for patients vs. healthy controls, respectively].

While the ANOVA for the error rate showed a significant main effect of compatibility [*F*_(1, 14)_ = 33.641, *p* < 0.001, η^2^ = 0.68], we also found a significant *group* × *compatibility* interaction [*F*_(1, 14)_ = 7.941, *p* = 0.014, η^2^ = 0.27] as the patients responded incorrectly more often in incompatible trials (23.6 ± 5.0 vs. 32.3 ± 5.1% in patients and 20.3 ± 5.3 vs. 23.5 ± 5.3% in healthy controls for compatible and incompatible trials, resulting in compatibility effects of 8.7 and 3.0% for patients and controls, respectively).

As demonstrated by the effect size (η^2^), the highest magnitude of differences was due to the factor *compatibility effect*, whereas the influence of the factor *group*, both in the analysis of RTs and the accuracy analysis, was quite moderate.

### Dual task

The RT data were analyzed by a three-factor analysis of variance (ANOVA) with *compatibility* and *muscle* as repeated measures variables and *group* as an independent factor. Once again, the main effect *group* [*F*_(1, 14)_ = 3.070, *p* = 0.100, η^2^ = 0.18] indicated a non-significant trend toward slower overall muscle activity among patients (mean ± standard error: 960 ± 61 and 837 ± 38 ms for patients and controls, respectively). The ANOVA also revealed a significant main effect of *muscle* [*F*_(1, 14)_ = 18.302, *p* = 0.001, η^2^ = 0.58] with the RTs of the zygomaticus major (mean ± standard error: 951 ± 42 ms) being slower in comparison to those of the corrugator supercilii (mean ± standard error: 846 ± 35 ms). The factor *group* did not influence this effect [*muscles* × *group* interaction *F*_(1, 14)_ = 0.023, *p* = 0.881, η^2^ = 0.01].

There was a compatibility effect of 18 ms [889 ± 37 vs. 907 ± 38 ms (mean ± standard error) for compatible and incompatible trials, respectively], but the corresponding main effect of *compatibility* [*F*_(1, 14)_ = 1.482, *p* = 0.244, η^2^ = 0.10] was not significant and was not influenced by the factor *group* [*group* × *compatibility* interaction; *F*_(1, 14)_ = 0.610, *p* = 0.809, η^2^ = 0.01]. The descriptive statistics showed a compatibility effect of 20 vs. 15 ms for patients and healthy controls, respectively, with the compatibility effect not reaching significance for either group (*t* = 1.607, *df* = 14, *p* = 0.130, and *t* = 0.697, *df* = 14, *p* = 0.497 for patients and healthy controls, respectively).

The ANOVA for error rates revealed no significant effects (all *F*s < 1). Patients responded incorrectly to 11.9 ± 5.4% of the incompatible and 10.0 ± 5.6% of the compatible trials. In the control group, error rates of 11.1 ± 5.9% for incompatible stimuli and 10.4 ± 5.6% for compatible stimuli were registered.

As the effect size (η^2^) reveals once again, the highest magnitude of differences was on account of the factor *muscle*, whereas the influence of the factor *group* was quite moderate, with the influence of the factor *compatibility effect* being negligible both in the accuracy analysis and analysis of RTs.

## Discussion

We investigated the effect of implicit processing of emotional facial expression on consciously controlled production of facial expressions (intentional imitation) in the group of stable patients with schizophrenia and healthy controls. In the first experiment (Simon task), we found a 60 ms-compatibility effect in reaction time (as measured by EMG muscle onset latency) triggered by the match (or mismatch) between the to-be-produced and simultaneously perceived facial expressions (see also Otte et al., 2011a). Both groups showed this effect, indicating intact spontaneous emotional expressions in patients. When the perceived facial expression was part of a different task (dual task), the compatibility effect was smaller (18 ms), falling short of the conventional significance threshold. The differences between our previous study (Otte et al., 2011b), in which a significant compatibility effect was seen among young healthy subjects, and the current work are likely to be explained by a relatively week influence of cross-task compatibility on the main task (intentional imitation) and a less homogeneous (in terms of age and education) group of control subjects in the current study.

To the best of our knowledge, this is the first study to investigate stimulated spontaneous emotional expressions in schizophrenia. While the study by Varcin et al. (2010) was conducted with the same objective, its approach, involving evaluation of the corrugator supercilii and zygomaticus major muscle responses during passive viewing of images of happy and angry facial expressions, was confounded by the generally reduced emotional expression (i.e., flat affect) in schizophrenia. Investigating stimulated spontaneous facial reactions, we, on the other hand, could show that more muted mimic reactions among schizophrenia patients are not necessarily affected by spontaneous facial mimicry or vice versa.

Reduced mimic emotional expression (i.e., flat affect) is a symptom of full-blown schizophrenia (Bleuler, 1950). It may be subtly manifested in emotional behavior before the onset of clinical symptoms (for review see Mandal et al., 1998) and increase following onset of illness (Walker et al., 1993). Our findings in stable patients corroborate the assumption that, rather than depending on the state of schizophrenic illness, decreased facial mimic reaction is likely to be a trait-like deficit. With regard to the activity of the corrugator supercilii and zygomaticus major muscles, we did not observe any differences between the groups, which likely indicates that deficits in mimic reaction are not necessarily emotion-specific. Underactivity of the laughter muscle (zygomaticus major muscle; Schneider et al., 1990), on the other hand, has been previously observed in schizophrenia. These discrepancies suggest that emotion-specific deficits are likely dependent on the state of schizophrenic illness. Indeed, acute schizophrenia patients or patients with more negative symptoms and/or chronic schizophrenia patients show a greater decoding deficit for negative compared to positive emotions (see Mandal et al., 1998).

In the dual task, we observed a more rapid activation of the corrugator supercilii in both groups, with response in the form of frowning being faster than smiling. Compared to the lower half of the face, the upper face is less voluntarily controlled (Rinn, 1984) and is subjected to an intuitive and spontaneous facial expression (Ekman and Friesen, 1978), resulting in more rapid activation in response to facial expressions. The presence of an affiliative function was suggested to result in a higher level of mimicry (at the level of the zygomaticus major) in response to happiness exhibited by an individual with a positive (compared to negative) social label (Beffara et al., 2012). This finding confirmed the function of mimicry theorized by Niedenthal et al. (2010), who postulated that the simulation of a facial expression activates brain areas involved in the construction of the expression's meaning on the basis of the subject's previous experience. Our results suggest that this function is unaffected in stable patients of schizophrenia. Given that the differences between stable patients and controls are subtle, only bigger cohorts of patients may render them appreciably apparent.

Finally, however, the results were limited by the small sample size of our study. In addition, patients' mimic reactions might also have been somewhat affected by medication, even though second generation antipsychotics are not known to have noteworthy effects on mimic reactions.

As already elucidated, we found a marginally weaker mimic reaction in stable patients with schizophrenia, which suggests a trait-like, rather than a state-dependent, characteristic of the deficits. In addition, in the Simon task, patients made considerably more mistakes in incongruent conditions. The observance of a conspecific's movement promptly activates our mirror neuron system, urging us to replicate the observed movement. However, because such automatic imitation is not always socially appropriate, an inhibitive component in our behavior seems crucial for effective social conduct (Bien et al., 2009). Thus, the higher number of mistakes in patients is likely indicative of the involvement of inhibitive components during automatic imitation. But with the differences in the compatibility effect being too subtle, drawing any conclusive inference is difficult.

Barring this effect, automatic encoding of facial expressions and spontaneous reaction to similar facial expressions were largely unaffected in patients. This observation, however, cannot be applied to a general sample of schizophrenia patients, and it remains unclear whether or not deficits in spontaneous mimic reaction occur in acute schizophrenia. It has been observed, for instance, that emotional functioning and recognition accuracy improve when patients suffering from psychosis move from an acute to a remitted phase (see Mandal et al., 1998). Whether deficits in spontaneous mimicry have state-dependent characteristics needs to be investigated in larger groups of patients, preferably at different stages of the disorder.

## Author contributions

Study conception and design: UH, IK. Acquisition of data: AP. Analysis and interpretation of data: AP, NC, EO. Drafting of manuscript: NC. Critical revision: IK, UH, NC, AP, EO.

## Funding

This research was funded by a grant from the Federal Ministry of Education and Research (BMBF 01GW0751).

### Conflict of interest statement

The authors declare that the research was conducted in the absence of any commercial or financial relationships that could be construed as a potential conflict of interest.
